# Functional Regions of Interest in Electrical Impedance Tomography: A Secondary Analysis of Two Clinical Studies

**DOI:** 10.1371/journal.pone.0152267

**Published:** 2016-03-24

**Authors:** Tobias Becher, Barbara Vogt, Matthias Kott, Dirk Schädler, Norbert Weiler, Inéz Frerichs

**Affiliations:** Department of Anesthesiology and Intensive Care Medicine, University Medical Center Schleswig-Holstein, Campus Kiel, Arnold-Heller-Str. 3, Haus 12, 24105, Kiel, Germany; University Children's Hospital Bern, SWITZERLAND

## Abstract

**Introduction:**

Patients with acute respiratory distress syndrome (ARDS) typically show a high degree of ventilation inhomogeneity, which is associated with morbidity and unfavorable outcomes. Electrical impedance tomography (EIT) is able to detect ventilation inhomogeneity, but it is unclear which method for defining the region of interest (ROI) should be used for this purpose. The aim of our study was to compare the functional region of interest (fROI) method to both the lung area estimation method (LAEM) and no ROI when analysing global parameters of ventilation inhomogeneity. We assumed that a good method for ROI determination would lead to a high discriminatory power for ventilation inhomogeneity, as defined by the area under the receiver operating characteristics curve (AUC), comparing patients suffering from ARDS and control patients without pulmonary pathologies.

**Methods:**

We retrospectively analysed EIT data from 24 ARDS patients and 12 control patients without pulmonary pathology. In all patients, a standardized low-flow-pressure volume maneuver had been performed and was used for EIT image generation. We compared the AUC for global inhomogeneity (GI) index and coefficient of variation (CV) between ARDS and control patients using all EIT image pixels, the fROI method and the LAEM for ROI determination.

**Results:**

When analysing all EIT image pixels, we found an acceptable AUC both for the GI index (AUC = 0.76; 95% confidence interval (CI) 0.58–0.94) and the CV (AUC = 0.74; 95% CI 0.55–0.92). With the fROI method, we found a deteriorating AUC with increasing threshold criteria. With the LAEM, we found the best AUC both for the GI index (AUC = 0.89; 95% CI 0.78–1.0) and the CV (AUC = 0.89; 95% CI 0.78–1.0) using a threshold criterion of 50% of the maximum tidal impedance change.

**Conclusion:**

In the assessment of ventilation inhomogeneity with EIT, functional regions of interest obscure the difference between patients with ARDS and control patients without pulmonary pathologies. The LAEM is preferable to the fROI method when assessing ventilation inhomogeneity.

## 1. Introduction

Electrical impedance tomography (EIT) is a non-invasive, radiation-free bed-side imaging modality that allows the assessment of regional ventilation distribution in patients suffering from acute respiratory distress syndrome (ARDS) [1, 2] and in healthy volunteers [3]. In the recent years, a growing interest in EIT as a tool for monitoring and guiding ventilator therapy has evolved [4]. Possible clinical applications of EIT imaging during mechanical ventilation are the real-time visualization of tidal recruitment [5–7], overdistension ([6, 7], changes in regional compliance [8–10] and inhomogeneity of regional ventilation distribution [11–14]. However, despite its increasing clinical use, some issues concerning the correct application of EIT remain unsolved.

**Fig 1:**
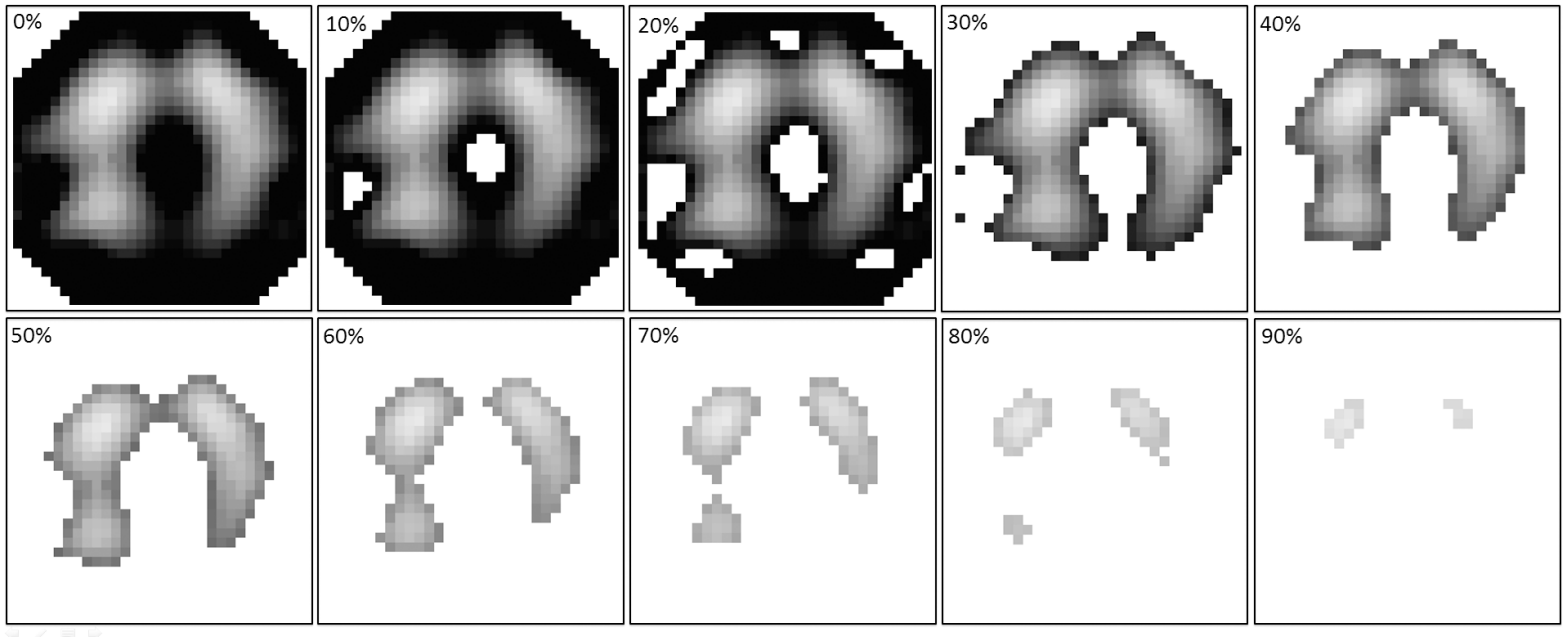
Functional regions of interest with different threshold criteria. The threshold criteria are expressed as percentages of the maximum impedance difference that was found during the low-flow maneuver. All image pixels with impedance differences below the threshold criterion are ignored during further image analysis.

One of these issues is the question whether or not a functional region of interest (fROI) should be used for EIT data analysis and which method for determining the fROI should be used preferably [15]. When an fROI is used, all EIT image pixels showing an impedance difference (ΔZ) below a predefined threshold value are ignored during further analysis. For example, with an fROI of 20% of the highest ΔZ in the image, all image pixels with a ΔZ smaller than 0.2 times the highest value in the image are ignored (Fig 1). This is a convenient way to exclude non-pulmonary soft tissues from the EIT image analysis. However, hypoventilated lung tissue and atelectatic lung areas are equally excluded from the EIT image analysis.

**Fig 2:**
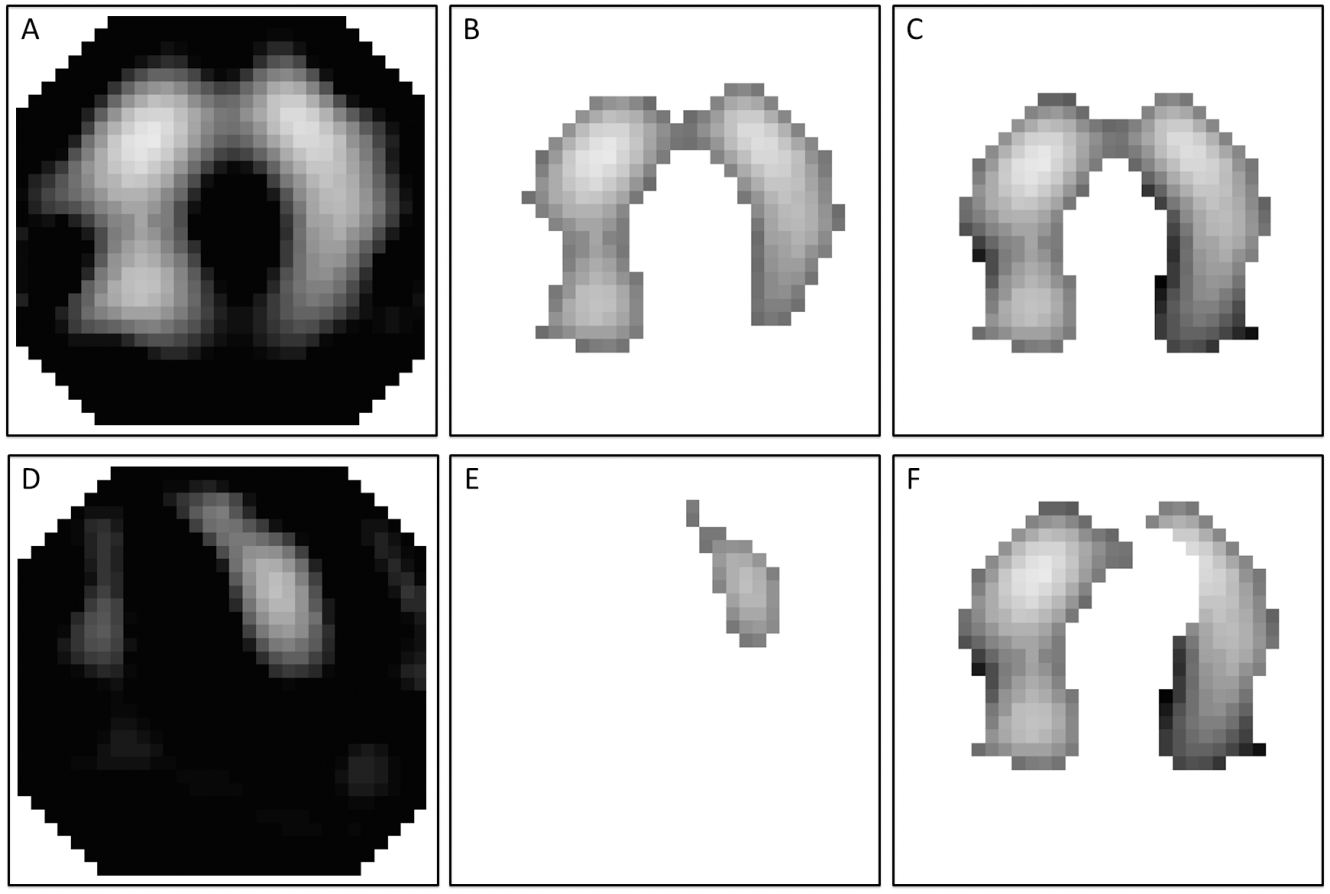
Creating an x-symmetrical region of interest (ROI) using the lung area estimation method (LAEM) in a patient with acute respiratory distress syndrome. From the raw image (A) a conventional functional ROI is created, in this example using a threshold criterion of 50% of the maximum impedance difference (B). In the next step (C), the ROI is mirrored by means of a Boolean “OR”-operation. The cardiac area is identified in the frequency domain (D, E) and subsequently subtracted from the x-symmetrical ROI to obtain the final LAEM ROI (F).

To partially overcome this limitation, the “lung area estimation method” (LAEM) was developed [11]. It is based on the assumption that in a healthy subject, the right and the left lung are relatively symmetrical. In brief, a conventional fROI is created in a first step and “mirrored” along the x-axis in a second step, creating a symmetrical ROI. In a third step, the cardiac area is identified in the frequency domain and subsequently subtracted from the x-symmetrical ROI (Fig 2).

Global measures of ventilation inhomogeneity like the global inhomogeneity index (GI index) or the coefficient of variation (CV) are highly sensitive to changes in fROI threshold criteria [11]. However, it remains unclear whether higher or lower fROI threshold criteria could improve the diagnostic accuracy of these measures and whether the conventional fROI method or the LAEM should be used when assessing ventilation inhomogeneity.

A high degree of ventilation inhomogeneity is typical in patients with ARDS [16]. Assuming a „normal“relatively symmetric lung shape, unilateral hypoventilated and atelectatic areas of the ARDS lung are included in the analysis with the LAEM, presumably leading to a higher value of the GI index and the CV. Using the fROI method, these hypoventilated or atelectatic areas are excluded, restricting the analysis to the relatively „healthy”parts of the lung. We therefore hypothesized that with respect to ventilation inhomogeneity, the LAEM would lead to a higher discriminatory power (as defined by the area under the receiver operating characteristics curve, AUC) between patients with ARDS and control patients without pulmonary disease.

The aims of our study were to asses the influence of the method of ROI determination on the discriminatory power of both the GI index and the CV between patients with ARDS and control patients, to investigate different threshold criteria both for the fROI and the LAEM, and to assess whether the GI index or the CV would lead to a higher discriminatory power between ARDS and control patients.

## 2. Methods

We retrospectively analyzed a database of 24 patients with ARDS and 12 control patients without pulmonary disease who had previously been enrolled in two clinical studies [1, 9]. Ethical approval for both studies was obtained from the ethics committee of the Christian Albrechts University Kiel, Germany. Written informed consent had been obtained from all patients or from their legal representatives. The studies were conducted in compliance with the Helsinki Declaration.

The patients were tracheally intubated and under controlled mechanical ventilation with an Evita XL ventilator (Dräger Medical, Lübeck, Germany). All patients underwent a standardized low-flow pressure-volume maneuver, starting at atmospheric pressure up to an airway pressure (P_aw_) of 35 mbar or a tidal volume (V_T_) of 2 l, at a constant flow of 4 l/min. Baseline patients characteristics are summarized in Table 1.

**Table 1:** Basic Patient characteristics.

	ARDS	Control	p (ARDS vs Control)
**Age (years)**	59 ± 15	46 ± 22	0.08
**Height (cm)**	175 ± 9	179 ± 9	0.18
**Weight (kg)**	79 ± 14	83 ± 12	0.37
**C**_**rs**_ **(ml/mbar)**	57 ± 20	88 ± 17	< 0.001
**Sex (m/f)**	17 m, 7 f	10 m, 2 f	not applicable

All values mean ± standard deviation. C_rs_: respiratory system compliance. ARDS: acute respiratory distress syndrome.

### 2.1 Acquisition of EIT data

EIT data were recorded with the Goe-MF II device (CareFusion, Yorba Linda, USA). 16 self-adhesive electrodes (Blue Sensor L-00-S, Ambu, Ballerup, Denmark) were placed around the chest circumference in one transverse plane lying approximately at the level of the fifth intercostal space. EIT images were obtained at a scan rate of 25 images per second.

### 2.2 Image reconstruction

Cross-sectional images representing the difference between maximum and minimum values of ΔZ during the low-flow maneuver were reconstructed from EIT data using a normalized difference reconstruction algorithm based on the Graz consensus reconstruction algorithm for EIT [17]. We refer to this image as a “max-min image”.

### 2.3 Regions of interest

All EIT images were analysed using the conventional fROI method and the LAEM.

In a first step, the maximum ΔZ was determined from the reconstructed EIT images. For this purpose, the lowest ΔZ value in the max-min image was subtracted from the highest ΔZ value in the image:

$$\Delta {\text{Z}}_{max}=Maximum(\Delta Z_{lung})-\;Minimum(\Delta Z_{lung})$$

The resulting value was subsequently multiplied with the fROI threshold criterion (fROI_thresh_) to yield the cut-off value for fROI determination (fROI_CutOff_):

$$fROI_{CutOff}=\Delta {\text{Z}}_{max}*fROI_{thresh}+Minimum(\Delta {\text{Z}}_{lung})$$

For the fROI method, all image pixels with ΔZ values higher than fROI_CutOff_ were used for the subsequent calculations (Fig 1).

For the LAEM, all pixels with ΔZ values above fROI_CutOff_ were subsequently mirrored by means of a Boolean “OR” operation (left to right and right to left) to yield an x-symmetrical ROI. The cardiac area was then identified in the frequency domain. To this end, a band pass filter that was adjusted 20% below and above the individual patient’s heart rate was applied to identify the cardiac-related impedance variations in every pixel. Subsequently, all pixels with impedance variations greater than 50% of the maximum cardiac-related impedance change were subtracted from the ventilation image to obtain the final lung area (Fig 2).

Since the removing the cardiac area is done for the LAEM but not for the “conventional” fROI method, we analyzed the influence of removing the cardiac area from the fROI separately. For this, the cardiac area, that was identified as described above, was subtracted from the fROI and all calculations were repeated using this “cardiac-depleted” fROI (fROI-heart).

### 2.4 Assessment of ventilation inhomogeneity

For the fROI method and the LAEM, we applied fROI_thresh_ values from 0.05 to 0.95 times ΔZ_max_ in steps of 0.05. For every fROI_thresh_, we calculated the GI index from all image pixels within the ROI according to [11]:

$$GI=\;\frac{\sum \left|\Delta {\text{Z}}_{xy}-Median\left(\Delta {\text{Z}}_{lung}\right)\right|}{\sum \Delta Z_{xy}}$$

where ΔZ_xy_ is the impedance difference between inspiration and expiration of an individual pixel in the ROI and ΔZ_lung_ are all pixel values of ΔZ in the ROI.

The CV was calculated from all image pixels in the ROI as the ratio of the standard deviation (SD) to the corresponding mean ΔZ value:

$$CV=\;\frac{SD(\Delta {\text{Z}}_{lung})}{Mean\left(\Delta {\text{Z}}_{lung}\right)}$$

Like the GI index, the CV was calculated for fROI_thresh_ values from 0.05 to 0.95 times ΔZ_max_ in steps of 0.05.

Additionally, both the GI index and the CV were calculated from all 912 EIT image pixels (“no fROI”).

### 2.5 Comparison of different methods and threshold criteria for ROI determination

All statistical analyses were performed using GraphPad Prism 6.0 (GraphPad Software, LaJolla California, USA).

In a first step, the number of pixels in the ROI was calculated for every fROI_thresh_ and was compared between ARDS and control patients using a two-sided unpaired t-test.

Subsequently, the mean values and 95% confidence interval (CI) of GI index and CV were calculated for all no fROI and all fROI_thresh_ criteria (fROI method and LAEM) both for ARDS and control patients.

For both the GI index and the CV, the AUC, 95% CI for AUC and p value for discrimination between ARDS and control patients were calculated using no fROI and for all fROI_thresh_ criteria applied to the fROI method and to the LAEM.

## 3. Results

### 3.1 Number of image pixels within the ROI

**Fig 3:**
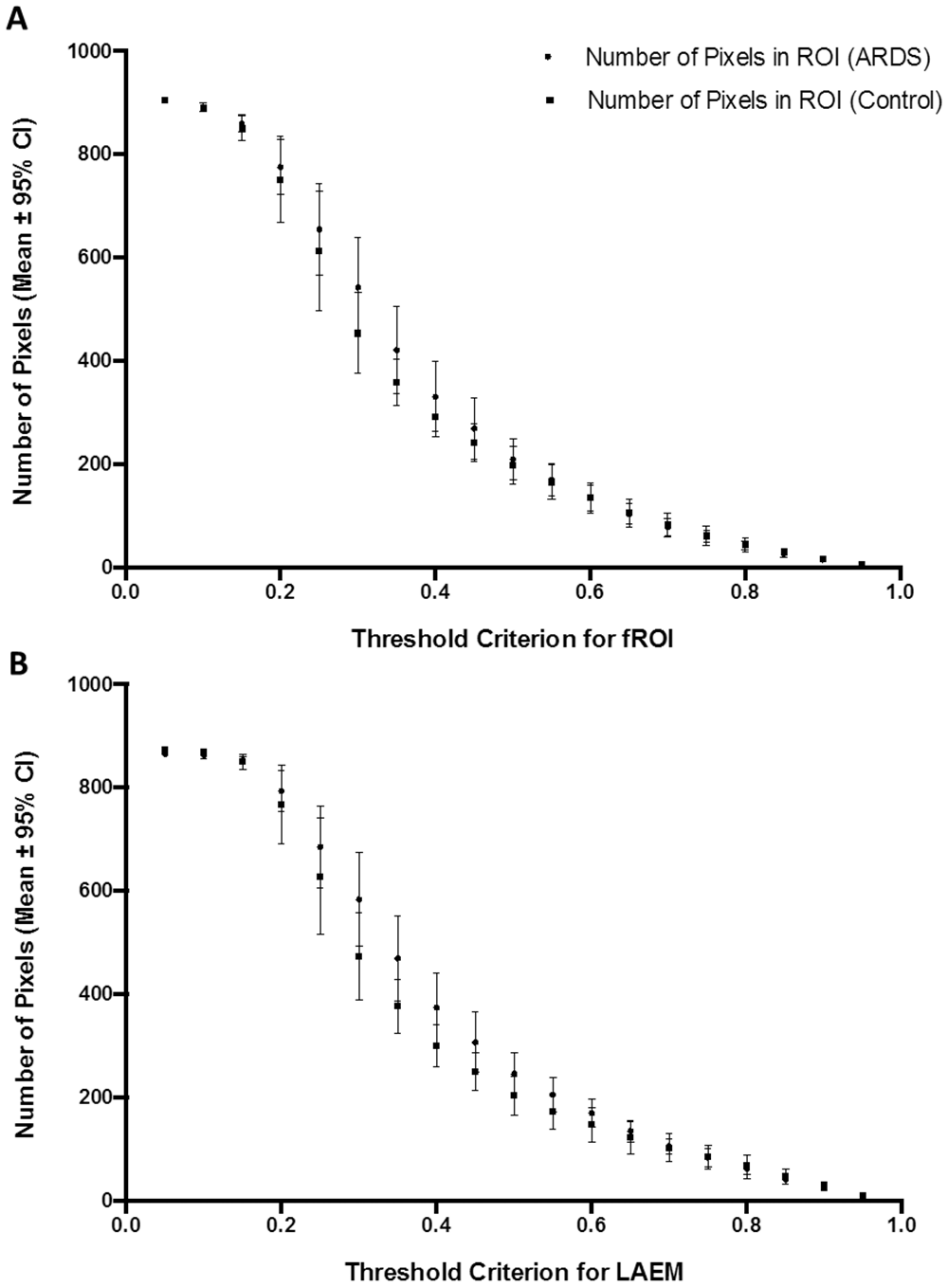
Number of pixels within the region of interest. (A) Number of image pixels within the functional region of interest (fROI) with increasing fROI threshold criteria. (B) Number of image pixels within the region of interest (ROI), determined using the lung area estimation method (LAEM), with increasing threshold criteria. No significant difference between patients with ARDS and control patients was observed with either method for ROI determination.

With the fROI method, we found a decrease in number of pixels with higher fROI threshold criteria both for ARDS and control patients. No significant difference in number of image pixels was observed between ARDS and control patients (Fig 3A).

With the LAEM, we found a similar decrease in number of image pixels with higher threshold criteria and no significant difference in number of image pixels between ARDS and control patients (Fig 3B).

### 3.2 Discrimination between ARDS and Control using no fROI

Analysing all 912 EIT image pixels, we found an AUC for discrimination between ARDS and control subjects of 0.76 for the GI index (95% CI for AUC = 0.58–0.94, p = 0.02) and of 0.74 for the CV (95% CI for AUC = 0.55–0.92, p = 0.02).

### 3.3 Discrimination between ARDS and Control using the fROI method

**Fig 4:**
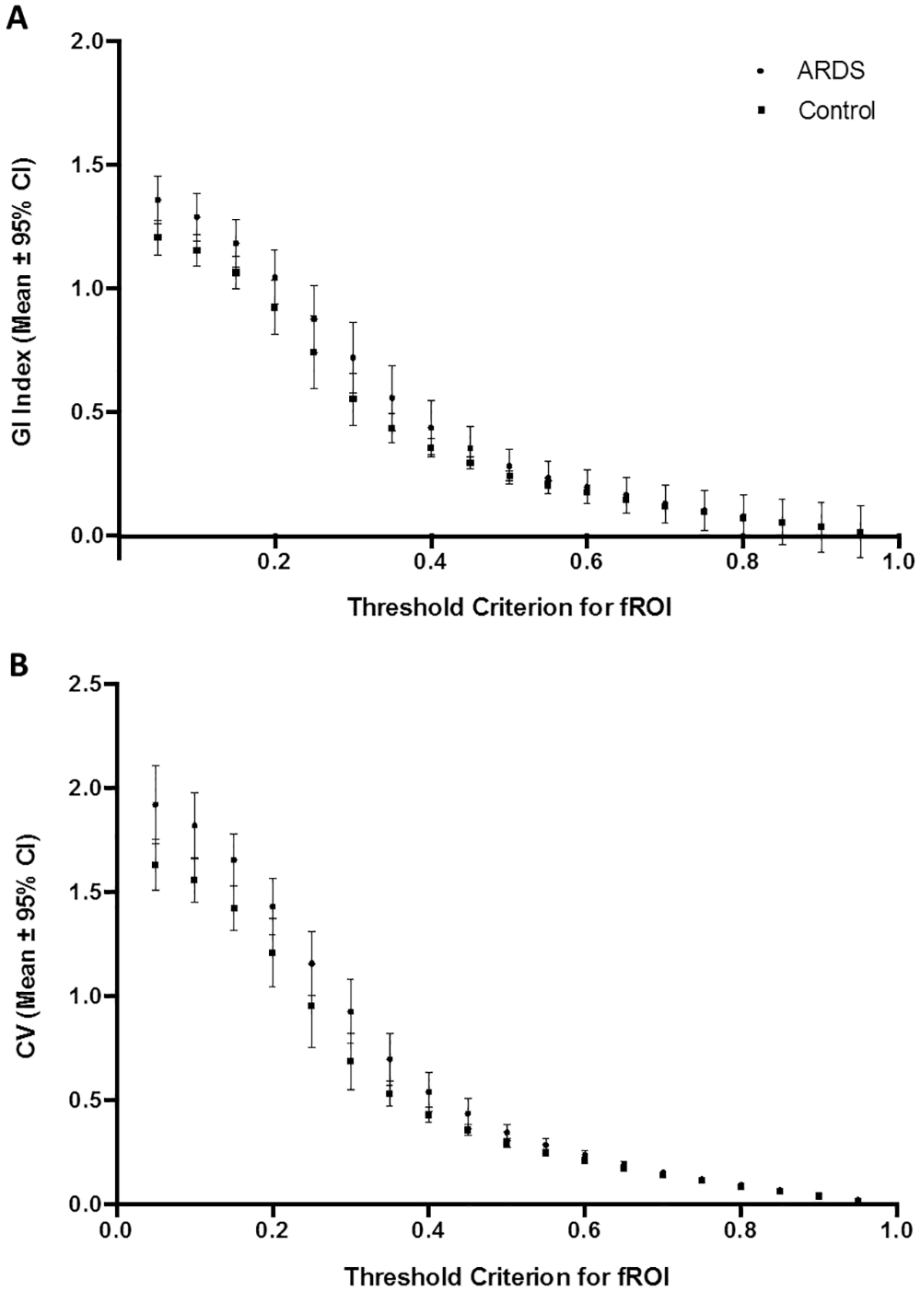
Threshold criterion for functional regions of interest. Effect of threshold criterion using the functional region of interest (fROI) method on the global inhomogeneity (GI) index (A) and on the coefficient of variation (CV) (B) in ARDS and control patients. * p < 0.05 for comparison between ARDS and control patients.

With increasing fROI threshold criteria, we found a decrease in GI index and CV both for ARDS and control patients. A significant difference between ARDS and control patients was observed with threshold criteria of 5%, 10%, 15% and 95% for the GI index (Fig 4A) and with threshold criteria of 5%, 10%, 15%, 20% and 95% for the CV (Fig 4B).

Using the fROI method, we found the best AUC for discrimination between ARDS and control patients with a fROI_thresh_ criterion of 10% both for the GI index (AUC = 0.74, 95% CI for AUC = 0.57–0.92, p = 0.02) and the CV (AUC = 0.74; 95% CI for AUC = 0.56–0.93, p = 0.02). With higher fROI_thresh_ criteria, we found a deteriorating AUC. All AUC values obtained using the fROI method are presented in Tables 2 (GI index) and 3 (CV).

**Table 2:** Area under the receiver operating characteristics curve (AUC), 95% confidence interval for AUC and p-value for different threshold criteria using functional region of interest (fROI) and the global inhomogeneity (GI) index. Comparison between patients suffering from acute respiratory distress syndrome (ARDS) and control patients without pulmonary pathologies.

fROI Threshold GI	AUC	95% CI	p (ARDS vs Control)
5%	0.74	0.56–0.92	0.02
10%	0.74	0.57–0.92	0.02
15%	0.73	0.56–0.90	0.03
20%	0.68	0.50–0.85	0.09
25%	0.67	0.49–0.85	0.10
30%	0.66	0.48–0.84	0.12
35%	0.65	0.47–0.84	0.14
40%	0.66	0.48–0.85	0.11
45%	0.68	0.50–0.86	0.08
50%	0.68	0.51–0.86	0.08
55%	0.67	0.50–0.85	0.09
60%	0.68	0.50–0.85	0.09
65%	0.70	0.53–0.87	0.06
70%	0.64	0.46–0.82	0.17
75%	0.55	0.36–0.74	0.61
80%	0.62	0.44–0.80	0.25
85%	0.60	0.42–0.79	0.31
90%	0.55	0.37–0.74	0.61
95%	0.71	0.54–0.89	0.04

**Table 3:** Area under the receiver operating characteristics curve (AUC), 95% confidence interval for AUC and p-value for different threshold criteria using functional region of interest (fROI) and the coefficient of variation (CV). Comparison between patients suffering from acute respiratory distress syndrome (ARDS) and control patients without pulmonary pathologies.

fROI Threshold CV	AUC	95% CI	p (ARDS vs Control)
5%	0.73	0.54–0.91	0.03
10%	0.74	0.56–0.93	0.02
15%	0.74	0.57–0.91	0.02
20%	0.74	0.58–0.91	0.02
25%	0.67	0.49–0.85	0.10
30%	0.67	0.49–0.85	0.10
35%	0.68	0.50–0.85	0.09
40%	0.68	0.50–0.86	0.08
45%	0.69	0.52–0.86	0.06
50%	0.67	0.49–0.84	0.11
55%	0.67	0.49–0.84	0.11
60%	0.64	0.46–0.82	0.16
65%	0.66	0.48–0.84	0.12
70%	0.63	0.45–0.81	0.20
75%	0.59	0.40–0.77	0.40
80%	0.63	0.45–0.81	0.20
85%	0.63	0.45–0.81	0.21
90%	0.50	0.31–0.69	1.00
95%	0.71	0.55–0.88	0.04

### 3.4 Discrimination between ARDS and Control using the lung area estimation method

**Fig 5:**
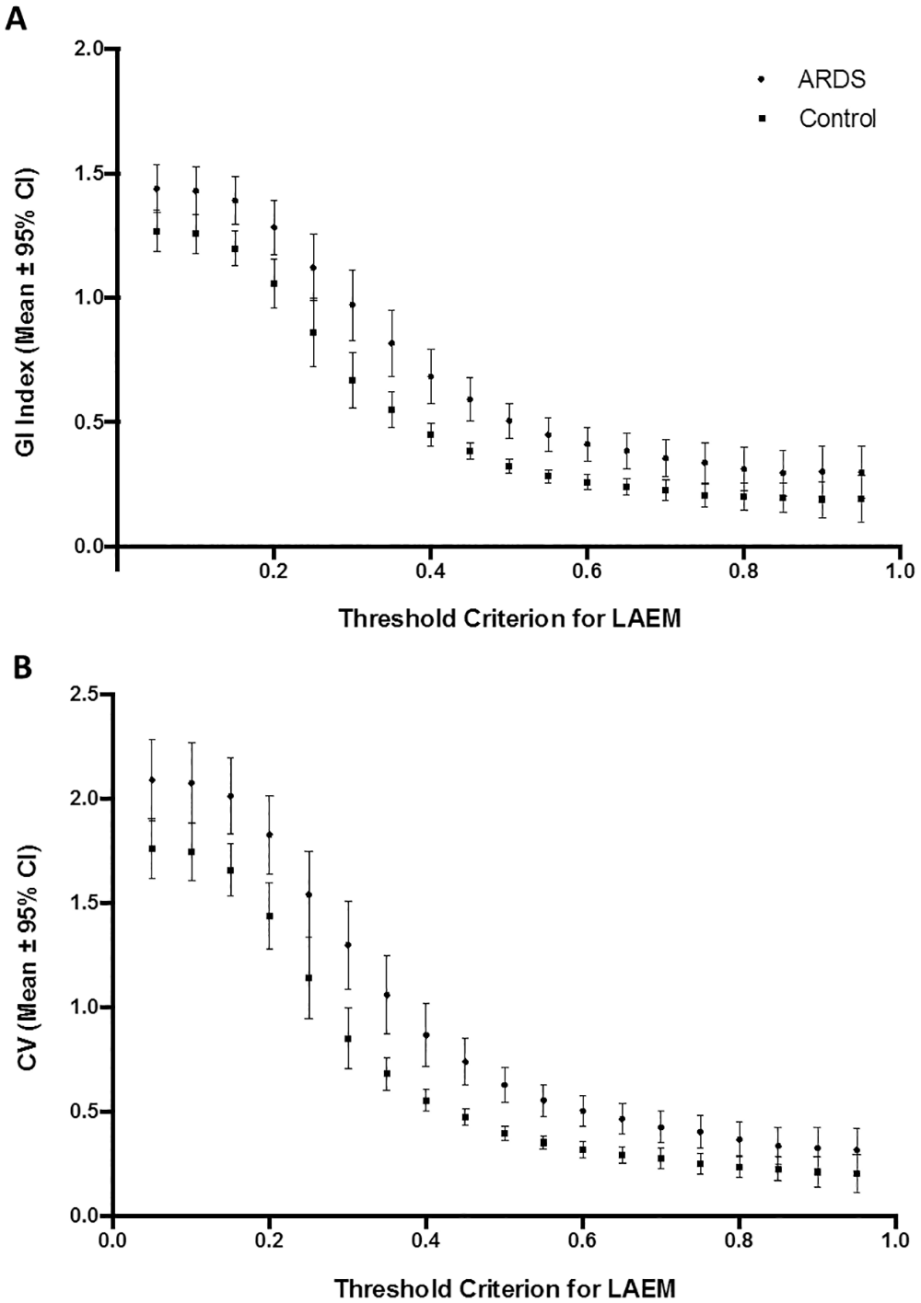
Threshold criterion for lung area estimation method. Effect of threshold criterion using the lung area estimation method (LAEM) on the global inhomogeneity (GI) index (panel A) and on the coefficient of variation (CV, panel B)in ARDS and control patients. * p < 0.05 for comparison between ARDS and control patients.

Similar to the fROI method, higher threshold criteria led to lower values of GI index and CV both for ARDS and control patients using the LAEM. A significant difference between ARDS and control subjects was found for all cut-off criteria between 5% and 75% of maximum ΔZ (Fig 5).

**Fig 6:**
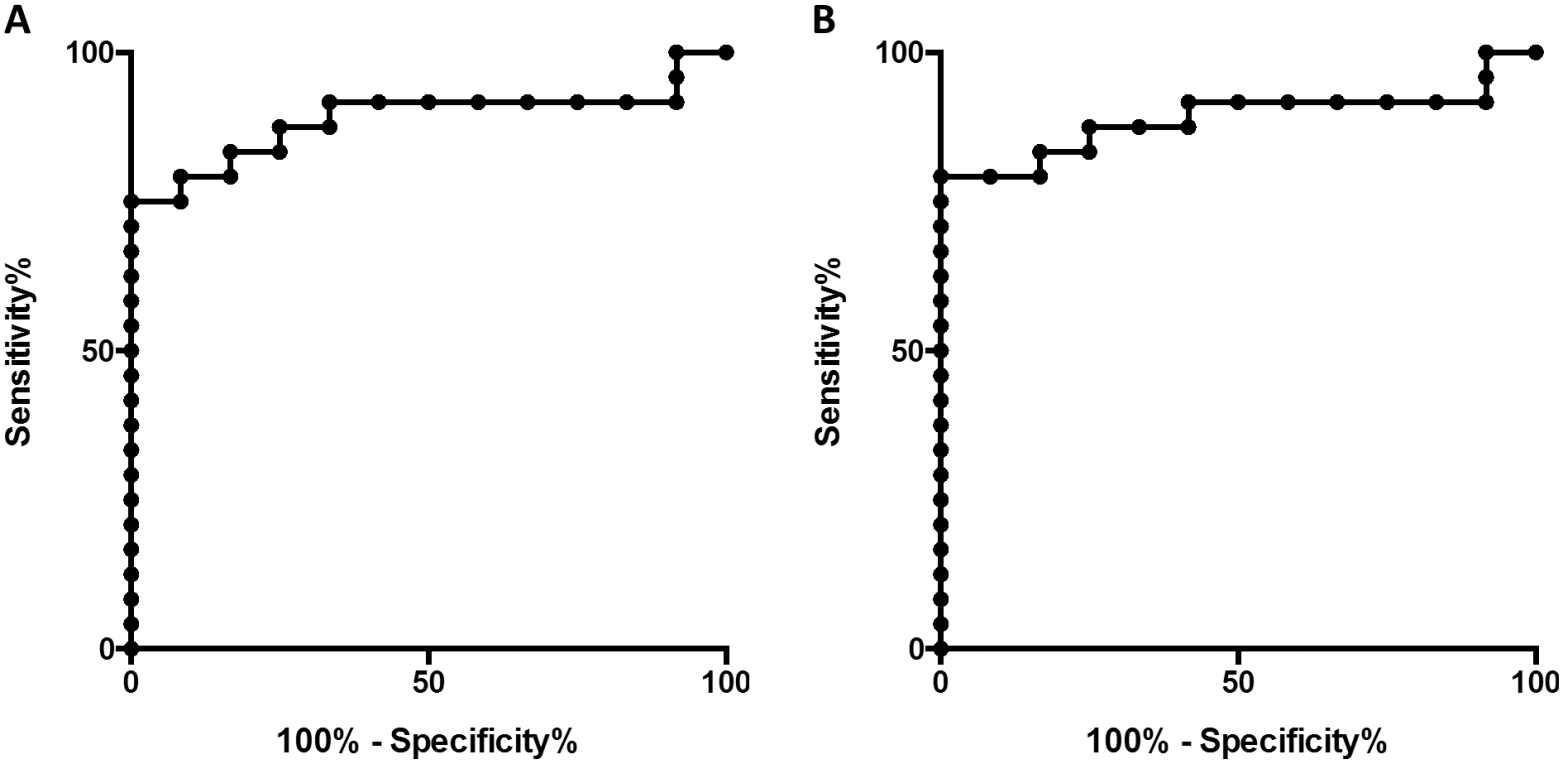
Discrimination between ARDS and control using the lung area estimation method. (A) Receiver operating characteristics (ROC) curve for discrimination between ARDS and control subjects using the global inhomogeneity index and the lung area estimation method with a criterion of 50% of the maximum impedance change. (B) ROC curve for the same comparison using the coefficient of variation.

Both for the GI index and the CV, an excellent discrimination between ARDS and control subjects was found with the LAEM at a threshold criterion of 50% (AUC = 0.89, 95% CI for AUC = 0.78–1.0, p < 0.001 for both indices; Fig 6). All AUC values obtained using LAEM for ROI determination for the GI index and CV are presented in Tables 4 and 5, respectively.

**Table 4:** Area under the receiver operating characteristics curve (AUC), 95% confidence interval for AUC and p-value for different threshold criteria using the lung area estimation method (LAEM) and the global inhomogeneity index (GI). Comparison between patients suffering from acute respiratory distress syndrome (ARDS) and control patients without pulmonary pathologies.

LAEM Threshold GI	AUC	95% CI	p (ARDS vs Control)
5%	0.79	0.62–0,95	0.006
10%	0.79	0.62–0.95	0.005
15%	0.77	0.61–0.93	0.009
20%	0.78	0.63–0.93	0.007
25%	0.75	0.59–0.91	0.017
30%	0.77	0.62–0.92	0.009
35%	0.79	0.64–0.94	0.005
40%	0.84	0.72–0.97	< 0.001
45%	0.86	0.74–0.98	< 0.001
50%	0.89	0.78–1.0	< 0.001
55%	0.89	0.78–1.0	< 0.001
60%	0.84	0.70–0.97	0.001
65%	0.81	0.67–0.95	0.003
70%	0.78	0.62–0.94	0.007
75%	0.74	0.57–0.90	0.021
80%	0.66	0.48–0.84	0.115
85%	0.62	0.44–0.80	0.240
90%	0.59	0.41–0,78	0.365
95%	0.61	0.42–0.80	0.283

**Table 5:** Area under the receiver operating characteristics curve (AUC), 95% confidence interval for AUC and p-value for different threshold criteria using the lung area estimation method (LAEM) and the coefficient of variation (CV). Comparison between patients suffering from acute respiratory distress syndrome (ARDS) and control patients without pulmonary pathologies.

LAEM Threshold CV	AUC	95% CI	p (ARDS vs Control)
5%	0.76	0.59–0.93	0.012
10%	0.76	0.59–0.93	0.012
15%	0.79	0.63–0.95	0.005
20%	0.79	0.65–0.94	0.005
25%	0.76	0.60–0.91	0.013
30%	0.77	0.62–0.92	0.009
35%	0.78	0.64–0.93	0.006
40%	0.85	0.73–0.98	< 0.001
45%	0.85	0.73–0.98	< 0.001
50%	0.89	0.78–1.00	< 0.001
55%	0.89	0.78–1.00	< 0.001
60%	0.85	0.73–0.98	< 0.001
65%	0.82	0.67–0.96	0.002
70%	0.79	0.64–0.94	0.005
75%	0.77	0.62–0.93	0.008
80%	0.69	0.52–0.86	0.064
85%	0.64	0.46–0.82	0.17
90%	0.62	0.44–0.81	0.23
95%	0.62	0.44–0.81	0.21

### 3.5 Effect of removing the heart region from the fROI

Removing the heart region from the fROI (fROI-heart) lead to no conceivable improvement in discriminatory power between ARDS and control patients both for the GI index (Table 6) and the CV (Table 7). The highest discriminatory power for fROI-heart was found with a threshold criterion of 5% for the GI index (AUC 0.76, 95% CI 0.58–0.93, p = 0.01) and of 10% for the CV (AUC 0.75, 95% CU 0.57–0.93, p = 0.02).

**Table 6:** Area under the receiver operating characteristics curve (AUC), 95% confidence interval for AUC and p-value for different threshold criteria using a functional region of interest with the heart region removed (fROI-heart) and the global inhomogeneity (GI) index. Comparison between patients suffering from acute respiratory distress syndrome (ARDS) and control patients without pulmonary pathologies.

fROI-heart Threshold GI	AUC	95% CI	p (ARDS vs Control)
5%	0.76	0.58–0.93	0.02
10%	0.75	0.57–0.93	0.02
15%	0.74	0.57–0.91	0.02
20%	0.68	0.50–0.86	0.08
25%	0.68	0.49–0.86	0.09
30%	0.66	0.50–0.86	0.08
35%	0.65	0.46–0.84	0.15
40%	0.66	0.47–0.84	0.11
45%	0.68	0.50–0.86	0.08
50%	0.68	0.51–0.86	0.08
55%	0.68	0.50–0.85	0.09
60%	0.64	0.46–0.82	0.18
65%	0.66	0.49–0.84	0.11
70%	0.60	0.42–0.78	0.33
75%	0.65	0.47–0.83	0.15
80%	0.67	0.50–0.85	0.09
85%	0.64	0.45–0.82	0.19
90%	0.56	0.37–0.75	0.57
95%	0.71	0.53–0.88	0.05

**Table 7:** Area under the receiver operating characteristics curve (AUC), 95% confidence interval for AUC and p-value for different threshold criteria using a functional region of interest with the heart region removed (fROI-heart) and the coefficient of variation (CV). Comparison between patients suffering from acute respiratory distress syndrome (ARDS) and control patients without pulmonary pathologies.

fROI-heart Threshold CV	AUC	95% CI	p (ARDS vs Control)
5%	0.75	0.57–0.92	0.02
10%	0.75	0.57–0.93	0.02
15%	0.73	0.56–0.91	0.02
20%	0.73	0.56–0.89	0.03
25%	0.69	0.52–0.87	0.06
30%	0.67	0.49–0.85	0.11
35%	0.66	0.48–0.84	0.12
40%	0.66	0.48–0.84	0.13
45%	0.67	0.49–0.85	0.10
50%	0.64	0.46–0.82	0.18
55%	0.64	0.46–0.82	0.18
60%	0.61	0.43–0.79	0.30
65%	0.64	0.46–0.82	0.17
70%	0.62	0.44–0.80	0.26
75%	0.65	0.47–0.83	0.16
80%	0.68	0.51–0.85	0.08
85%	0.65	0.47–0.83	0.15
90%	0.60	0.41–0.78	0.35
95%	0.69	0.52–0.87	0.07

## 4. Discussion

The goal of this research was to make a contribution to establishing EIT as a tool for recognition of patients suffering from ARDS based on ventilation inhomogeneity. Therefore, we selected two established methods for ROI definition, the fROI method and the LAEM, and used them in the assessment of the two most frequently used EIT parameters for ventilation inhomogeneity, the GI index and the CV. Our main findings are that the method of ROI definition has a high influence on the ability of EIT to discriminate between ARDS and control patients, whereas the choice of GI index or CV seemed to have no influence. The best area under the curve was identified for the LAEM using a threshold value of 50%.

Ventilation inhomogeneity has been investigated in several studies assessing patients with EIT. In the first study analysing the GI index using the LAEM for ROI determination, Zhao et al. found a significant difference in GI index between patients with diseased lungs scheduled for thoracic surgery and healthy control patients, as well as a significant difference between two-lung ventilation and one-lung ventilation [11]. Again using the LAEM for ROI determination during an incremental and decremental PEEP trial in 10 ventilated patients without pulmonary pathology, Zhao et al. found no significant difference between “best PEEP” derived from the dynamic compliance and from the GI index method [12].

Using the fROI method, Blankman et al. [18] found no difference in GI index between pressure controlled ventilation and pressure support ventilation in patients after cardiac surgery. During a decremental PEEP trial from 15 to 0 in 4 steps of 5 mbar, the lowest GI index was identified at levels of 15 and 10 mbar using the same method for ROI determination [19].

Using no fROI, we found a significant difference in CV between patients suffering from chronic obstructive pulmonary disease (COPD) and healthy control subjects during pulmonary function testing [20] and a significantly lower CV in COPD patients during high frequency oscillatory ventilation in comparison to conventional mechanical ventilation [14].

### 4.1 Comparison between no fROI, fROI and LAEM

Our findings indicate that the fROI method impairs the discriminatory power between ARDS and control patients both for the GI index and for the CV. To this end, analysing all EIT image pixels (no fROI), removing the heart region at most, seems to be a better strategy.

The LAEM is a further development of the fROI method, designed to overcome its intrinsic limitations when analysing ventilation inhomogeneity in a lung with hypoventilated or atelectatic areas. Our results indicate that this improves the discriminatory power between ARDS and control patients. This may be due to the fact that in most patients with ARDS, the spatial distribution of hypoventilated and atelectatic areas is not identical between the right and left lung. Therefore, the “mirroring” along the x axis that is part of the LAEM, can be helpful in identifying these impaired lung areas.

However, in a patient with a relatively symmetrical distribution of hypoventilated and atelectatic areas in the right and left lung, the LAEM would also lead to an underestimation of ventilation inhomogeneity. Therefore, advanced developments like anatomical regions of interest, based on CT scans of patients with comparable height and weight, could further improve the assessment of ventilation inhomogeneity [21].

In general, the results obtained for the GI index and the CV were very similar, indicating that both indices are equally effective in discriminating between ARDS and control patients with respect to ventilation inhomogeneity.

### 4.2 Influence of ROI threshold criterion

As illustrated in Fig 4, the fROI method leads to a progressive loss in discriminatory power between ARDS and healthy control patients with increasing threshold criteria. The same finding applies to the fROI method when the heart region is removed.

For the LAEM, we found the best discriminatory power between healthy patients and ARDS patients with a fROI_thresh_ of 0.5 times ΔZ_max_. This finding implies that 50% could be a useful threshold criterion for discrimation between healthy and diseased lungs. At first sight, this appears to be a very high threshold criterion. It is important to realize that ΔZ_max_ was calculated as the difference between maximum(ΔZ_lung_) minus minimum(ΔZ_lung_) and not as maxium(ΔZ_lung_) minus zero. Negative values of ΔZ_lung_ were found in all patients. Out-of-phase impedance changes are common during ventilation [22] and, when ignored, can affect the outcome of ROI definition significantly. If ΔZ_max_ was simply calculated as maximum(ΔZ_lung_) minus zero, much more pixels would be ignored with the same threshold criterion.

### 4.3 Clinical significance

In patients with ARDS, ventilation inhomogeneity, as assessed by computed tomography (CT), is associated with overall disease severity and mortality [16]. An intervention that decreases ventilation inhomogeneity could therefore, theoretically, result in improved patient outcomes. However, repeated CT scans are not feasible for assessing the results of therapeutic interventions and would result in excessive radiation exposure for the patient.

EIT has the potential to assess ventilation inhomogeneity at the bedside without any radiation exposure. Titrating positive end-expiratory pressure (PEEP) according to EIT-derived indices of ventilation inhomgeneity is feasible [12]. Nevertheless, an incorrect application of EIT for minimizing ventilation inhomogeneity is potentially dangerous.

Our results highlight the large influence of ROI criteria on EIT indices of ventilation inhomogeneity. According to our results, the LAEM seems to be superior to the fROI method when assessing ventilation inhomogeneity in ARDS and control patients. Analysing all 912 image pixels with no fROI seemed to be superior to the fROI method but inferior to the LAEM when trying to discriminate between ARDS and control patients.

### 4.4 Risk of optimization of ventilator settings aiming for ventilation homogeneity

In healthy lungs, some inhomogeneity in ventilation distribution is physiologic. Adjustment of ventilator settings aiming only for the highest possible ventilation homogeneity could therefore be misleading or even deleterious [23]. Normal values of GI index and CV are yet to be defined under close consideration of the method of ROI definition.

One must also bear in mind that during a PEEP trial, the same pixels could be related to other lung tissue due to lung recruitment or collapse, which would affect the pixel-based analysis that is used for calculation of GI index and CV. In these cases, the results of GI index and CV might be misleading.

### 4.5 Limitations

Our study has some important limitations. First, it was a retrospective analysis of EIT data which had been recorded in two different studies. However, the similar study protocol and the standardized low-flow pressure-volume maneuver make these data comparable.

Second, we limited our analyses to the low-flow pressure-volume maneuver and did not analyse normal tidal ventilation. This was, in part, due to the retrospective nature of our analyses. We decided to analyse the low-flow pressure-volume maneuver because it is a standardized maneuver that, in our study, always started at a P_aw_ of 0 mbar up to a maximum P_aw_ of 35 mbar or a maximum V_T_ of 2 l. Since both P_aw_ and V_T_ have a significant influence on the GI index [12, 24], we chose this standardized maneuver for our data analysis to make the data on ventilation inhomogeneity inter-patient comparable. However, it is unclear to what extent our data can be extrapolated to patients undergoing normal tidal ventilation without a low-flow pressure-volume maneuver.

To our knowledge, a „golden standard”for assessment of ventilation inhomogeneity with EIT has not yet been defined. The aim of our manuscript was to compare the fROI method and the LAEM when using the GI index and the CV to discriminate between ARDS and healthy patients. These two indices have often been used to quantify ventilation inhomogeneity in studies from different EIT research groups. Whether our findings can be extrapolated to other methods for assessment of ventilation inhomogeneity remains to be established.

Another limitation of our study is the relatively small sample size of 24 ARDS and 12 control patients. A replication of our results in a larger prospective study is desirable.

### 4.6 Conclusion

In the assessment of ventilation inhomogeneity with EIT, functional regions of interest may obscure the difference between patients with ARDS and control patients without pulmonary pathologies. When analysing ventilation inhomogeneity with a standardized low-flow pressure-volume-maneuver, it is therefore preferable to use the lung area estimation method or to analyse the whole EIT image.

Standardization of EIT data acquisition and analysis is of paramount importance for the successful interpretation of EIT-derived measures [25]. In any case, the method of ROI definition should therefore be reported in all papers dealing with EIT.
